# Evaluation of an *in silico *predicted specific and immunogenic antigen from the OmcB protein for the serodiagnosis of *Chlamydia trachomatis *infections

**DOI:** 10.1186/1471-2180-8-217

**Published:** 2008-12-10

**Authors:** Olfa Frikha-Gargouri, Radhouane Gdoura, Abir Znazen, Boutheina Gargouri, Jalel Gargouri, Ahmed Rebai, Adnene Hammami

**Affiliations:** 1 Department of Microbiology and research laboratory "Microorganismes et Pathologie Humaine", Habib Bourguiba hospital of Sfax, Tunisia; 2 Department of blood bank, Sfax, Tunisia; 3 Bioinformatics Unit, Centre of Biotechnology of Sfax, Tunisia

## Abstract

**Background:**

The OmcB protein is one of the most immunogenic proteins in *C. trachomatis *and *C. pneumoniae *infections. This protein is highly conserved leading to serum cross reactivity between the various chlamydial species. Since previous studies based on recombinant proteins failed to identify a species specific immune response against the OmcB protein, this study evaluated an *in silico *predicted specific and immunogenic antigen from the OmcB protein for the serodiagnosis of *C. trachomatis *infections.

**Results:**

Using the ClustalW and Antigenic programs, we have selected two predicted specific and immunogenic regions in the OmcB protein: the N-terminal (Nt) region containing three epitopes and the C-terminal (Ct) region containing two epitopes with high scores. These regions were cloned into the PinPoint Xa-1 and pGEX-6P-1 expression vectors, incorporating a biotin purification tag and a glutathione-S-transferase tag, respectively. These regions were then expressed in *E. coli*. Only the pGEX-6P-1 has been found suitable for serological studies as its tag showed less cross reactivity with human sera and was retained for the evaluation of the selected antigens. Only the Ct region of the protein has been found to be well expressed in *E. coli *and was evaluated for its ability to be recognized by human sera. 384 sera were tested for the presence of IgG antibodies to *C. trachomatis *by our in house microimmunofluorescence (MIF) and the developed ELISA test. Using the MIF as the reference method, the developed OmcB Ct ELISA has a high specificity (94.3%) but a low sensitivity (23.9). Our results indicate that the use of the sequence alignment tool might be useful for identifying specific regions in an immunodominant antigen. However, the two epitopes, located in the selected Ct region, of the 24 predicted in the full length OmcB protein account for approximately 25% of the serological response detected by MIF, which limits the use of the developed ELISA test when screening *C. trachomatis *infections.

**Conclusion:**

The developed ELISA test might be used as a confirmatory test to assess the specificity of serological results found by MIF.

## Background

*Chlamydiaceae*, intracellular obligate bacteria, is divided into two genera *Chlamydia *and *Chlamydophila*. The only species infecting humans in the genus *Chlamydia *is *Chlamydia trachomatis*, the most common cause of genital tract infections. The genus *Chlamydophila *contains six species. *Chlamydophila pneumoniae *and occasionally, *Chlamydophila psittaci *and *Chlamydophila abortus *[1,2] (also called *Chlamydophila psittaci *serovar1) are human pathogens. *C. pneumoniae *and *C. psittaci *cause respiratory tract infections whereas *C. abortus *causes abortion. While serology is the current method of choice in routine clinical laboratories for the diagnosis of acute *C. pneumoniae *infections [3], nucleic acid amplification tests [4,5] represent the methods of choice for the diagnosis of acute *C. trachomatis *infections. The usefulness of serology in the diagnosis of *C. trachomatis *infections has been reviewed [6,7]. It is generally accepted that serology is used for the serodiagnosis of complications in ascending chlamydial infections and in seroepidemiological studies [8-10].

Several methods were used for the serological diagnosis of chlamydial infections. The microimmunoflurescence (MIF) test, which detects antibodies to chlamydial elementary bodies, has long been considered to be the gold standard for the serodiagnosis of chlamydial infections. However, this method lacks standardization. Furthermore, cross reactivity between the chlamydial species, which affects the specificity of this test, was reported in the literature [11-13]. These cross reactions between the chlamydial species would, therefore, hamper the diagnosis and the interpretation of chlamydial serology. Several enzyme linked immunosobent assays (ELISA) have been developed. Promising results were obtained with these tests using as antigen purified chlamydial elementary bodies devoided of the genus specific lipopolysaccharide (LPS) [14-16]. However, serum cross reactivity persisted due to the presence of other genus specific epitopes as well as residual contaminants. The use of chemically or recombinant antigens seems to be attractive to improve chlamydial serodiagnosis. This approach requires the identification of the most immunodominant antigens in human *C. trachomatis *infections. Proteomic and immunoblot analyses demonstrated that the most immunogenic chlamydial components comprise the genus specific LPS, the major outer membrane protein (MOMP) and the OmcB protein (also called outer membrane protein 2, OMP2) [17-23]. The MOMP protein contains 4 surface exposed variable domains that are the major sites of antigenicity [24]. The variable domains I, II and IV contain the primary serovar determining epitopes, allowing *C. trachomatis *classification into serovars [25].

The OmcB protein is the second most abundant outer membrane protein in *Chlamydiae*. The *omcB *gene of *C. trachomatis *comprises 1641 bp and encodes a 60 kDa protein. The translated amino acid sequence reveals a relatively basic protein containing 24 cysteine residues [26]. It has been suggested that disulphide cross linked polymers of the OmcB protein are the functional equivalent of peptidoglycan in other Gram negative bacteria, forming a disulphide cross linked network with the periplasmic domains of MOMP and other membrane proteins which may contribute to the considerable structural stability of *C. trachomatis *elementary bodies [27]. Although this protein is a major immunogen in chlamydial infections, it was shown to be localized at the inner surface of the outer membrane complex [28]. In fact, *in vivo *as a result of host defence reactions during the course of the infection, determinants of previously inaccessible bacterial proteins may be released from disintegrated cell walls and become exposed to B cell antigen receptors. Furthermore, upon protein unfolding after limited proteolysis, previously inaccessible linear motifs can be exposed to serve as B cell epitopes and become immunogenic [29]. Recently, Fadel *et al*. [30] suggested that the OmcB protein of *C. trachomatis *is surface exposed and that it functions as a chlamydial adhesion protein since anti-OmcB antibody inhibits *in vitro *infectivity of *C. trachomatis*.

The OmcB protein is highly conserved among the chlamydial species [31] so that serum cross reactivity with the OmcB of *C. pneumoniae *is frequently seen [29,32,33]. In order to identify a species specific humoral immune response directed against *C. trachomatis *OmcB protein, Mygind *et al*. [33] prepared three overlapping fusion proteins and found that immunodominant linear epitopes recognized by human antibodies were distributed throughout the entire sequence of the OmcB protein. In the current study, we used internet accessible computer based algorithms to assess their ability to predict OmcB species specific epitopes recognized in human chlamydial infections. Using the ClustalW and Antigenic programs, we have selected two predicted specific and immunogenic regions [34,35]. These regions were cloned into the PinPoint Xa-1 and pGEX-6P-1 expression vectors incorporating a biotin purification tag (BPT) and a glutathione-S-transferase tag (GST), respectively, to facilitate the purification of the recombinant proteins. Our main objective was to develop an ELISA test based on an *in silico *predicted specific and immunogenic antigen from the OmcB protein for the serodiagnosis of *C. trachomatis *infections and to evaluate the antigen using a considerable number of sera. Furthermore, we aimed at assessing the ability of the two vectors to express recombinant fusion proteins detecting species specific antibodies to *C. trachomatis *without or with minimal reactivity with tags.

## Methods

### Bioinformatics

#### Alignments of OmcB proteins of *C. trachomatis*, *C. pneumoniae *and *C. psittaci *and determination of percentage identity

OmcB protein sequences of *C. trachomatis *serovars as well as *C. pneumoniae *and *C. psittaci *were retrieved from the NCBI database in FASTA format. The Needle program was used to perform global pair wise alignments of sequences for determining percentage identities between OmcB proteins and/or peptides . *C. trachomatis *serovar E was used as the reference sequence for all alignments performed. The ClustalW program was used to construct multiple sequence alignment using the default parameters . Accession numbers of protein sequences used of *C. trachomatis *serovars, *C. pneumoniae *and *C. psittaci *are shown in table 1.

**Table 1:** Percentage identities determination between the OmcB proteins, the Nt and Ct regions within the chlamydial species.

**Chlamydial species**	**Serovars**	**Protein accession numbers**	**OmcB protein**	**Nt region **	**Ct region**
*C. trachomatis*	A	REFSEQ: YP_328263	97.5% (539/553)	18.3% (101/553)	7.2% (40/553)
	B	EMBL: CAA37588	98.5% (539/547)	18.5% (101/547)	7.3% (40/547)
	C	GenBank: AAA23159	98.7% (540/547)	18.5% (101/547)	7.3% (40/547)
	D	REFSEQ: NP_219955	97.5% (539/553)	18.3% (101/553)	7.2% (40/553)
	E	EMBL: CAA39396	100% (547/547)	19.0% (104/547)	7.3% (40/547)
	F	GenBank: AAA23154	99.1% (542/547)	18.6% (102/547)	7.3% (40/547)
	G/H/K	Swiss-Prot: Q548P6	98.5% (539/547)	18.5% (101/547)	7.3% (40/547)
	I/J	Swiss-Prot: Q933I7	98.5% (539/547)	18.5% (101/547)	7.3% (40/547)
	L1/L2/L3	Swiss-Prot: P21354	97.8% (535/547)	18.3% (100/547)	7.3% (40/547)

*C. pneumoniae*		EMBL: CAA37590	71.5% (398/557)	8.8% (49/557)	4.3% (24/556)

*C. psittaci*		EMBL: CAA37592	70.9% (397/560)	8.2% (46/560)	4.7% (26/557)

Nt: N-terminal, Ct: C-terminal, Percentage identities were determined using the Needle program. *C. trachomatis *serovar E was used as the reference sequence for all alignments performed.

#### Antigenicity prediction

Antigenicity prediction was performed using the Antigenic program of the Pasteur institute . This program predicts potentially antigenic regions of a protein, using the method of Kolaskar and Tongaonkar [36].

#### Determination of the molecular weight

The molecular weights (Mw) of the selected regions were determined by the computer pI/Mw tool , [37].

### Generation of recombinant proteins

#### Bacterial strains, plasmids and media

*C. trachomatis *serovar E was used as a template for PCR reaction. *Escherichia coli *strain Top 10 was used as a cloning host. The *E. coli *strain BL21 (DE3), which contains the structural gene for T7 RNA polymerase under the control of the lac promoter, was used for protein expression. The pMOS blue blunt ended cloning kit (Amersham) was used as a cloning vector. The plasmids PinPoint Xa-1 (Promega), and pGEX-6P-1 (Amersham) under the control of the tac promoter, were used as expression vectors. The PinPoint Xa-1 vector places the insert fragment in frame with an N terminal region encoding a polypeptide of 123 amino acid fusion tag that becomes biotinylated in *E. coli *by an endogenous biotin ligase activity resulting in the addition of a single biotin to lysine at residue 88 of the polypeptide. The pGEX-6P-1 expression vector places the insert fragment in frame with an N terminal region encoding the GST. *E. coli *strains were grown in Luria Bertani (LB) medium, supplemented with 100 μg/ml ampicillin when plasmid maintenance was required. In the case of the biotinylated fusion proteins, expressed using the PinPoint Xa-1 vector, 2 μM of biotin were also included in the medium.

#### DNA preparation of *C. trachomatis*

Chlamydial DNA was extracted using an in house method based on proteinase K treatment. Briefly, 100 μl of the cultured *C. trachomatis *serovar E was centrifuged at 14000 rotations per minute (rpm) for 30 minutes. The supernatant was discarded and the pellet was washed twice with phosphate buffered saline (PBS). The DNA was released with proteinase K (Invitrogen) in lysis buffer (10 mM Tris-HC1 pH 8, 1 mM EDTA). Proteins were digested at 55°C for 1 hour. The enzymatic reaction was then stopped by incubation at 98°C for 10 minutes.

#### Cloning and construction of the overexpression plasmids

The parts of the *omcB *gene, encoding the selected parts of the OmcB protein by the bioinformatics tools, were amplified by PCR from genomic DNA of *C. trachomatis *serovar E. The PCR mixture, which was made up to 50 μl with sterile water, contained 1× PCR buffer (50 mM Tris-HCl pH 8.3, 10 mM KCl, 5.0 mM (NH_4_)_2_SO_4_, and 2.0 mM MgCl_2_); 0.5 mM of each primer; 0.2 mM each dATP, dCTP, dGTP and dUTP; 1.25 U of the high fidelity *Pfu *DNA polymerase (Promega, France); and 5 μl of prepared DNA extract from *C. trachomatis *serovar E. PCR was performed using the Gene-Amp PCR System 9700 (Perkin Elmer Cetus) under the following conditions: an initial cycle at 95°C for 5 minutes, followed by 35 cycles of denaturation at 94°C for 30 seconds, annealing at 55°C for 30 seconds, and elongation at 72°C for 30 seconds, with a final cycle at 72°C for 7 minutes.

For cloning of the Nt region in the PinPoint Xa-1 vector, the phosphorylated amplification product was cloned into the pMOS blue blunt ended cloning kit. The amplification product using the forward and U19 primers introduces a *Bam*HI site that is used to generate a 3' cohesive end in order to direct the cloning of the insert in the PinPoint Xa-1 vector. Thus, the amplification product was digested by *Bam*HI, purified, phosphorylated and then ligated to a previously *Nru*I/*Bam*HI linearised and purified PinPoint Xa-1 vector.

For cloning of the Ct region in the PinPoint Xa-1 vector, the amplification product was purified, phosphorylated and ligated to a previously *Nru*I linearised, purified and dephosphorylated PinPoint Xa-1 vector. The correct orientation of the insert in the vector was verified by amplification using the reverse and T7 primers.

For cloning in pGEX-6P-1 expression vector, the PCR products were double digested by *Eco*RI/*Not*I or *Bam*HI/*Not*I according to the cloned region. The double digestion products were purified and ligated into a previously *Eco*RI/*Not*I or *Bam*HI/*Not*I linearised and purified vector according to the cloned region, respectively. All enzymes for molecular cloning were obtained from Invitrogen, and assay conditions were made according to the manufacturer's instructions.

The ligation mixtures were transferred into *E. coli *Top10 competent cells using the CaCl_2 _method. The presence of the appropriate insert was determined by PCR and by restriction analysis. DNA products were analysed on a standard 2% agarose gel containing ethidium bromide (Sigma). DNA sequences were elucidated by the dideoxynucleotide chain termination method according to a cycle sequencing protocol using thermosequenase (Amersham Pharmacia Biotech) with the DNA sequencer ABI PRISM 3100/3100-Avant Genetic Analyser. The identified positive colonies were grown in LB medium containing ampicillin (100 μg/ml), and the recombinant plasmids were isolated from bacteria cells using a plasmid extraction kit (Promega). The isolated plasmids were then used to transform *E. coli *strain BL21 (DE3) competent cells for expression purposes.

#### Expression of recombinant proteins

The *E. coli *BL21 (DE3) cells harbouring the recombinant plasmids were grown at 37°C, to an optical density (OD) of 0.6 at 600 nm, in 5 ml LB medium containing 100 μg/ml ampicillin. In the case of the biotinylated fusion proteins expressed using the PinPoint Xa-1 vector, 2 μM of biotin were also included in the medium. The culture medium was then adjusted to 1 mM isopropyl-β-D-thiogalactopyranoside (IPTG) and incubation continued at 37°C for 3 hours.

For large scale expression, cells were grown in 500 ml of the culture media in the same conditions as the small scale expression, harvested by centrifugation at 6000 rpm for 10 minutes and washed twice with PBS. Bacteria harbouring recombinant PinPoint Xa-1 plasmids, collected by centrifugation, were resuspended in cell lysis buffer (50 mM Tris-HCl pH 7.5, 50 mM NaCl, 5% glycerol, 5 mM phenyl-methyl-sulfonyl-fluoride (PMSF) and 1 mM benzamidine), those harbouring recombinant pGEX-6P-1 plasmids, collected by centrifugation, were resuspended in PBS. The cells were sonicated to release intracellular proteins and the extract was then centrifuged at 12000 rpm for 20 minutes to remove cell debris.

#### Purification of the recombinant proteins

Two methods of purification of the biotinylated fusion proteins expressed using the PinPoint Xa-1 vector were used. These proteins were first purified by affinity chromatography using the SoftLink™ Soft Release Avidin Resin (Promega) according to the manufacturer's instructions. The clear supernatant was mixed with 1 ml of the resin equilibrated with cell lysis buffer for 2 hours at room temperature or overnight at 4°C with gentile agitation. The crude extract-resin was then centrifuged at 800 g for 3 minutes, the supernatant was eliminated and the resin was thoroughly washed with cell lysis buffer. Proteins were then eluted with 5 mM biotin in the same buffer. The second method of purification consisted in heating the extract, obtained after cell lysis and centrifugation, at 70°C for 5 minutes. The denatured proteins were then eliminated by centrifugation at 12000 rpm for 20 minutes.

For the purification of proteins expressed using the pGEX-6P-1 vector, the clear supernatant was mixed with 1 ml of the glutathione beads (Sigma) equilibrated with PBS for 30 minutes at room temperature. The crude extract-glutathione beads were then centrifuged at 800 g for 3 minutes, the supernatant was eliminated and the resin was then washed twice with 6 ml PBS. Proteins were then eluted with 5 mM oxidized glutathione (Sigma) in PBS.

Purified protein concentration was determined as described by Bradford [38] using bovine serum albumin (BSA) as standard.

#### Polyacrylamide gel electrophoresis and Western blot analysis

Polyacrylamide gel electrophoresis of proteins (SDS-PAGE) was performed in the presence of sodium dodecyl sulfate (SDS) (0.3 M) and β-mercaptoethanol (0.25 M), as described by Laemmli [39]. Proteins were dissolved in SDS sample buffer and boiled for 2 minutes. These proteins were separated by SDS-PAGE in 15% gels containing 3% stacking gel and transferred to a nitrocellulose membrane by electroblotting. The membrane was stained with ponceau S in order to visualize the protein markers and blocked with the TBST buffer (10 mM Tris-HCl, pH 8.0, 150 mM NaCl, 0.05% Tween 20) for 1 hour at 37°C. The membrane was then incubated, under gentile agitation, for 30 minutes at 37°C with streptavidin alkaline phosphatase (Promega), to detect the biotinylated fusion proteins expressed using the PinPoint Xa-1 expression vector. The membrane was washed three times with TBST for 5 minutes and rinsed briefly with deionized water. Finally, the biotinylated proteins were visualized after staining with western blue stabilized substrate for alkaline phosthatase (Promega) for 30 minutes.

### Serology

#### Sera

A set of 244 sera was assembled, comprising 24 sera from patients that were MIF positive to only *C. trachomatis *antigens, 72 sera from a high risk population of prostitutes, 112 sera from healthy blood donors and 44 sera from patients referred to sexually transmitted infection (STI) clinics suspected to have chlamydial infections diagnosed by Cobas Amplicor test as PCR positive (n = 14) or PCR negative (n = 30). In addition, a set of 52 sera from MIF positive cases to *C. pneumoniae *and a control set of 52 children, aged from 2 to 8 years whose sera were *C. trachomatis *and *C. pneumoniae *MIF negative, were also included to produce a panel of 356 sera. All these sera were tested by MIF and ELISA. Only 18 sera were tested by western blot analysis including 7 sera from healthy blood donors, 2 sera from prostitutes, 7 sera from patients MIF *C. pneumoniae *positive, 1 serum from patients MIF *C. trachomatis *positive and 1 serum from patients *C. trachomatis *PCR negative. All the subjects provided verbal informed consent, and the study protocol was approved by our ethics committee (Association d'Enregistrement et de Lutte Contre le Cancer du Sud Tunisien).

#### Serological methods

##### MIF

Chlamydial IgG antibodies were determined as previously described by Wang and Grayston [40]. *C. trachomatis *and *C. pneumoniae *species specific IgG antibodies were measured by our in house MIF test using purified elementary bodies of *C. pneumoniae*, IOL-207 strain, *C. psittaci *Loth strain and *C. trachomatis *L2 strain, as antigens. These antigens were produced in yolk sac membranes of infected eggs. The sacs of uninfected eggs were used as negative control. Slides were prepared as acetone fixed preparations of the purified antigens by experienced laboratory technicians capable of maintaining all conditions equal between test runs. The antigen densities for all experiments were guaranteed by an optimal concentration of elementary bodies. Sera were tested in serial twofold dilutions for IgG from 1/16 to the end point in order to determine their IgG antibody titers. All MIF series included a positive and a negative serum. Incubation time was 30 minutes with diluted sera and 30 minutes with 1:300 fluorescein isothiocyanate (FITC) conjugated anti-human immunoglobulin (biorad) in a moisture chamber at 37°C. After each of these incubations, the slides were washed twice for 5 minutes with PBS. The mounting fluid for setting coverslips on the slides contained glycerol in PBS buffer. All the slides were examined by two experienced and independent readers using a fluorescent microscope (Zeiss AxioStar Plus) with × 40 objective. In case of discordant readings, the slides were assessed by a third reader. Results were interpreted using the same microscope and by the same experienced readers in the same period.

##### Western blot analysis using human sera

After electrophoresis and transfer, the membrane was cut into strips. These strips were blocked with the blocking buffer (PBS supplemented with 5% dried milk powder) and incubated with human sera as primary antibodies diluted at 1:100 in blocking buffer for an additional hour at 37°C. The secondary antibody (Horseradish Peroxydase (HRP)-conjugated rabbit anti-human IgG (Dako)) was diluted 1:1000 and incubated with the strips for 1 hour at 37°C. The membranes were washed three times with washing buffer (PBS, 0.05% tween 20) after each of these incubations. Finally the strips were washed twice with PBS and developed in the dark for 30 minutes with 0.07% 4-chloro-1-naphtol (Sigma) and 0.01% H_2_O_2 _in PBS. The development was stopped by replacing the development solution by distilled water.

##### ELISA

Maxisorp microliter 96 well ELISA plates (Nunc) were coated with 60 μl of the antigen solution per well (4 μg/ml) in PBS and incubated overnight at 4°C. The coating solution was discarded before the ELISA plates were blocked with 75 μl of 3% BSA in PBS and incubated for 1 hour at 37°C. The plates were washed three times with PBS containing 0.05% tween 20 (washing buffer). Fifty μl per well of serum samples diluted 1:50 in PBS were added in duplicate and incubated for 1 hour at 37°C. In the same way, 50 μl per well of the secondary antibody (Horseradish Peroxydase (HRP)-conjugated rabbit anti-human IgG (Dako)), diluted 1:10000, were added and incubated for additional 1 hour at 37°C. Between each of these incubations, the wells were washed three times with washing buffer using a plate washer. The reaction was visualized with 50 μl tetramethyl benzidine (TMB) substrate per well and incubated for 30 minutes at 37°C in the dark. The development was stopped by adding 100 μl of 1 M HCl solution per well. The results were read immediately by photometric readings of the OD at 450 nm with a reference wavelength of 620 nm.

### Statistics

All data were collected using standardized forms and were analyzed by Epi-Info version 6 and SPSS version 11.

## Results

### Bioinformatics research

The percentage identities determination of the OmcB proteins of *C. trachomatis *serovars and those of *C. pneumoniae *and *C. psittaci *according to *C. trachomatis *serovar E are presented in table 1. Using the Needle program, the OmcB protein of *C. trachomatis *serovar E, used as the reference sequence, was found to share 97% identity with the OmcB proteins of *C. trachomatis *serovars A, D, L1, L2 and L3; 98% identity with those of serovars B, C, G, H, I, J and K; 99% identity with that of serovar F and 100% identity with that of serovar E. Lower identities were found with the OmcB proteins of *C. pneumoniae *(71%) and *C. psittaci *(70%).

**Figure 1:**
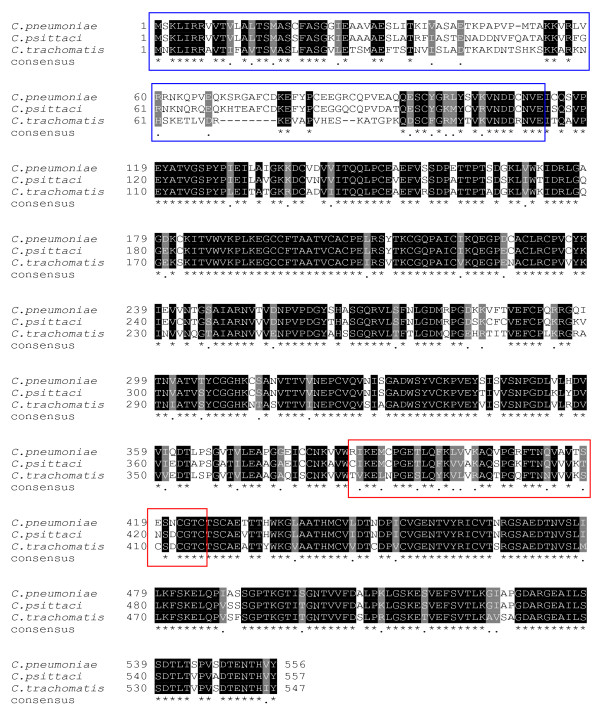
**Multiple sequence alignment of the OmcB proteins of *C. trachomatis*, *C. pneumoniae *and *C. psittaci***. The multiple sequence alignment was performed using the ClustalW program. The selected N-terminal and C-terminal regions, which represent a lesser degree of similarity, are boxed. Stars mark identical amino acid residues in all sequences in the alignment. Dashes represent gaps in the alignment introduced by the ClustalW program. Conserved amino acids are shaded dark grey and physico-chemical conserved amino acids are shaded in light grey.

The alignment of the OmcB sequences using the ClustalW program helped us identify two regions conserved within *C. trachomatis *serovars but varying between the chlamydial species. The two regions are localized in the N-terminal (Nt) and C-terminal (Ct) parts of the OmcB protein of *C. trachomatis *spanning amino acids 1 to 104 and 377 to 417, respectively (fig 1).

Using the Needle program and the Nt region of the OmcB protein of *C. trachomatis *serovar E as the reference sequence, 9% and 8% identities were found with the OmcB proteins of *C. pneumoniae *and *C. psittaci*, respectively; 19% identity with the OmcB proteins of *C. trachomatis *serovar E and F, and 18% with those of all the remaining serovars. Using the same program, the Ct region of *C. trachomatis *serovar E was found to share 7% identity with all the OmcB proteins of *C. trachomatis *serovars, 4% and 5% identities with the OmcB proteins of *C. pneumoniae *and *C. psittaci*, respectively.

The immunogenicity analysis using the Antigenic program indicated that the epitopes of the OmcB protein are distributed throughout the protein (See additional file 1). The selected Nt region contains 3 epitopes which are not highly immunogenic according to their scores in the full length OmcB protein antigenicity prediction classified in the 14^th^, 19^th ^and 24^th ^positions. The selected Ct region contains only 2 epitopes that are positioned at the third and 6^th ^places according to their scores in the full length OmcB protein antigenicity prediction.

The predicted molecular weights of the Nt and Ct regions were about 11.5 and 5.5 kDa, respectively.

### Cloning of the selected Nt and Ct regions and expression of the recombinant proteins

The two selected regions by the bioinformatics tools were successfully amplified and cloned into the PinPoint Xa-1 and pGEX-6P-1 expression vectors using the appropriate restriction enzymes (table 2).

**Table 2:** Primer sequences, size and location of DNA fragments in the *omcB *gene.

**Vector**	**Region**	**Primer^a^**	**Sequences^b^**	**Size of DNA fragment (bp)**	**Location of DNA fragment (bp)^c^**
**PinPoint Xa-1**	Nt	F R	CCCTACGAACAAACTCATCAGAC AACAAGCTTTTATTCAACATTACGATCA	312	1–312
		U19	GTTTTCCCAGTCACGTTGTA		
	Ct	F R	^§^**CGA**GACTGTAAAGAACTGAATCCTG AATAAGCTTTCAACAGGTACCACAGTCA	120	1131–1251
		T7	TTGTGAGGGGATAACAATTTC		
	S	F R	GTGACGCGGTGCAGGGCG ATTTAGGTGACACTATAG		

**pGEX-6P-1**	Nt	F R	^£^ACCC**GAATTC**CCAATGAACAAACTGATAAGAC ^#^AAC**GCGGCCGC**TTATTCAACATTACGATCA	312	1–312
	Ct	F R	^¥^AGAC**GGATCC**TGGACTGTGAAAGAACTG ^#^AAT**GCGGCCGC**TCAACAGGTACCACAGTCA	120	1131–1251
	S	F R	CCAGCAAGTATATAGCATGG CCGGGAGCTGCATGTGTCAGAGG		

^a^: F, forward, R, reverse, S, sequencing primer. ^b^: Listed 5'-3'. The sequences in bold are primers restriction enzymes used for cloning (^§ ^:*Nru*I, ^£^: *Eco*RI,^#^: *Not*I, ^¥^:*BamH*I). ^c^: Locations of DNA fragments are according to Genbank accession number [EMBL: X55903].

The recombinant plasmids were transferred to *E. coli *BL21 (DE3) for expression purposes. In total, 4 recombinant plasmids were produced encoding 4 proteins: the BPT-Nt and the BPT-Ct expressed using the PinPoint Xa-1 vector, the GST-Nt and the GST-Ct expressed using the pGEX-6P-1 vector, respectively for the Nt and Ct regions of the OmcB protein. The expected molecular weights of these four proteins were 24.5, 18.5, 35.5 and 29.5 kDa, respectively. The SDS-PAGE and western blotting results showed a concordance between the molecular weights of these proteins and those expected by the bioinformatic tool.

**Figure 2:**
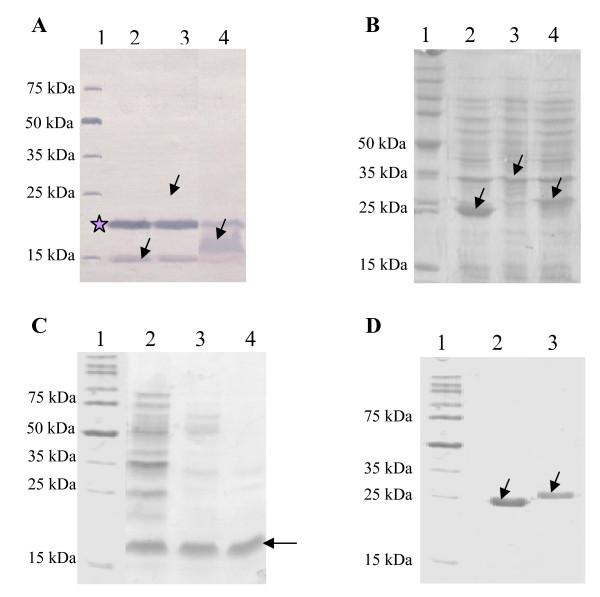
**Expression and purification of the recombinant proteins and the vector tags**. All proteins of interest are indicated with arrows. (A): Immunoblot analysis of the BPT tagged proteins expressed using the PinPoint Xa-1 vector in *E. coli *BL21 (DE3). Lane 1: molecular weight marker (broad range protein molecular marker, PROMEGA), lane 2: BPT (13 kDa), lane 3: BPT-Nt (24.5 kDa), lane 4: BPT-Ct (18.5 kDa). The star marks an endogenous biotinylated protein of 22.5 kDa expressed in *E. coli *cells. (B): SDS-PAGE (15%) analysis of GST tagged proteins expressed using the pGEX-6P-1 vector in *E. coli *BL21 (DE3). Lane 1: molecular weight marker, lane 2: GST (24 kDa), lane 3: GST-Nt (35.5 kDa), lane 4: GST-Ct (29.5 kDa). (C): SDS-PAGE (15%) analysis of the semi purified BPT-Ct by heat treatment. Lane 1: molecular weight marker, lane 2: 40°C 5 min, lane 3: 55°C 5 min, lane 4: 70°C 5 min. (D): SDS-PAGE (15%) analysis of purified GST and GST-Ct. Lane 1: molecular weight marker, lane 2: purified GST, lane 3: purified GST-Ct.

The expression level of the Nt region was too low using the two vectors as shown in figure 2A and 2B. Furthermore, the presence of these recombinant proteins was not reproducible. On the contrary, the expression levels of the BPT-Ct and the GST-Ct were high using the two vectors (fig 2A and 2B). However, the recombinant proteins expressed using the PinPoint Xa-1 vector were quickly degraded even in presence of protease inhibitors.

The expression of the tags alone using the native vectors was also investigated. As found with the BPT-Nt, the BPT expression was too low and detectable only by western blot (fig 2A). On the contrary, the GST was highly expressed (fig 2B).

Since the expression of the Nt region failed to generate visible and reproducible proteins, the two constructs were abandoned and all further steps were made only with the Ct region.

### Purification of the Ct recombinant proteins and the vector tags

All attempts made to purify the BPT-Ct and the BPT according to the manufacturer's recommendations failed. A simple heat treatment of the extract at 70°C for 5 minutes allowed the purification of the BPT-Ct to nearly 80% (fig 2C). The BPT was also purified using the same protocol; however the purified BPT band was not visible in SDS-PAGE but reactive in western blot. No further reactive bands were observed by western blot analysis of the BPT and the BPT-Ct using human sera.

Proteins expressed using the pGEX-6P-1 vector were purified efficiently using glutathione beads affinity chromatography (fig 2D). 1100 μg of the GST-Ct and 28000 μg of the GST have been obtained from 500 ml of the culture of the positive colonies.

### Assessment of the reactivity of the BPT-Ct, the GST-Ct and the vector tags by western blot

**Figure 3:**
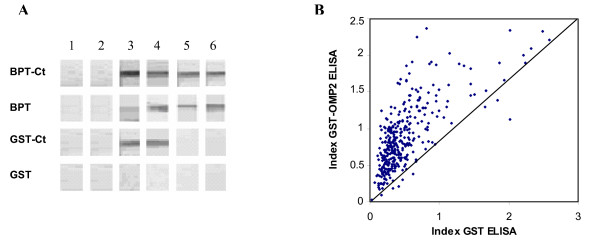
**Immunoreactivity of the recombinant proteins and the vector tags**. (A): Immunoblot analysis of the BPT-Ct, the BPT, the GST-Ct and the GST using 6 human sera. Lane 1: purified BPT-Ct, lane 2: purified BPT, lane 3: purified GST-Ct, lane 4: purified GST. Each column represents the reactivity of one serum to the four antigens. Column 1 and 2: sera from healthy blood donors, column 3: serum from prostitute, column 4, 5 and 6: sera from MIF *C. pneumoniae *positive patients. (B): ELISA results using the GST and the GST-Ct as antigens obtained with the 356 sera. Each dot represents the GST-Ct reactivity according to that of the GST for the same serum.

First, we investigated the reactivity of the OmcB Ct region by western blot analysis using human sera. We, therefore, included the purified tags as negative controls for each serum. The reactivity of six sera to the BPT-Ct, the GST-Ct and the vector tags alone is shown in figure 3A. Positive sera to the Ct region reacted equally with the BPT-Ct and the BPT. However for the GST-Ct, the sera did not show any reactivity with the GST. The western blot results for the 18 tested sera are summarised in table 3. The GST generated less reactivity with human sera than the BPT. Furthermore, the GST and the GST-Ct were purified to homogeneity. Consequently, the GST-Ct was retained for its evaluation by ELISA using the 356 sera.

**Table 3:** Immunoblot results of the Ct recombinant proteins related those of the vector tags.

**Vector**		**Tag-Ct reactivity/Tag reactivity**			
**PinPoint Xa-1**	No. of sera	BPT-Ct+/BPT+	BPT-Ct +/BPT-	BPT-Ct -/BPT+	BPT-Ct -/BPT-
		12	0	0	6

**pGEX-6P-1**	No. of sera	GST-Ct+/GST+	GST-Ct+/GST-	GST-Ct-/GST+	GST-Ct-/GST-
		2	2	0	14

BPT: biotin purification tag expressed using the native PinPoint Xa-1 vector, BPT-Ct: the C-terminal (Ct) recombinant protein expressed using the PinPoint Xa-1 vector, GST: glutathione-S-transferase expressed using the native pGEX-6P-1 vector, GST-Ct: the C-terminal (Ct) recombinant protein expressed using the pGEX-6P-1 vector.

### Assessment of the reactivity of the GST-Ct and the GST by ELISA

In order to assess the GST reactivity in ELISA and its effect on the GST-Ct ELISA results, all sera were subjected to a double ELISA using as antigens the GST and the GST-Ct. The reactivity of each serum to each antigen was tested in duplicate. Figure 3B shows the reactivity of the GST-Ct according to that of the GST for the same serum. All sera showed level of reactivity to the GST ranging from 0.03 to 2.58. So, the reactivity of each serum to the OmcB Ct region was determined by the formula: net OD value of the OmcB Ct ELISA = [mean OD value of the purified GST-Ct] – [mean OD value of the purified GST alone].

### Evaluation of the OmcB Ct antigen by ELISA

#### Reproducibility of the OmcB Ct ELISA test

**Figure 4:**
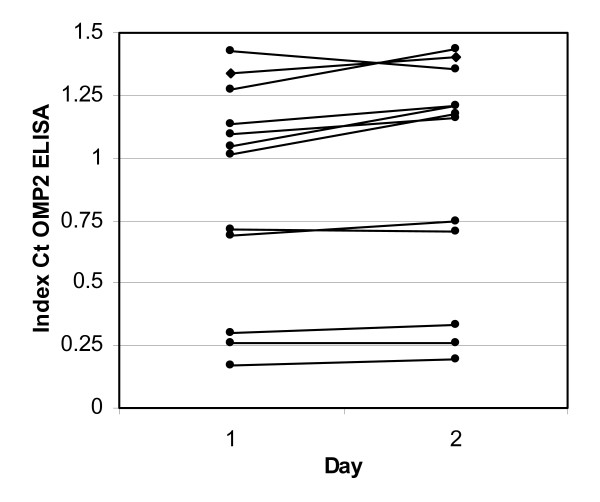
**Reproducibility of the OmcB Ct ELISA assay**. The figure shows OD values, at 450 nm, of the 12 tested sera obtained in two ELISA tests conducted on two different days. Each line indicates the reactivity of one serum on the two days. The differences between the OD values for all sera were less than 20%.

The reproducibility of the ELISA test was determined by analyzing 12 sera with low, medium and high reactivity to the OmcB Ct region, run under the same conditions on different days. The difference between the OD values for each serum was lower than 20% (fig 4).

#### Determination of the cut off value of the OmcB Ct ELISA test

Using sera from the healthy blood donors, the cut off value, calculated by the formula: mean OD value + two standard deviations, was found to be 0.764.

#### Correlations between the MIF and OmcB Ct ELISA tests

**Figure 5:**
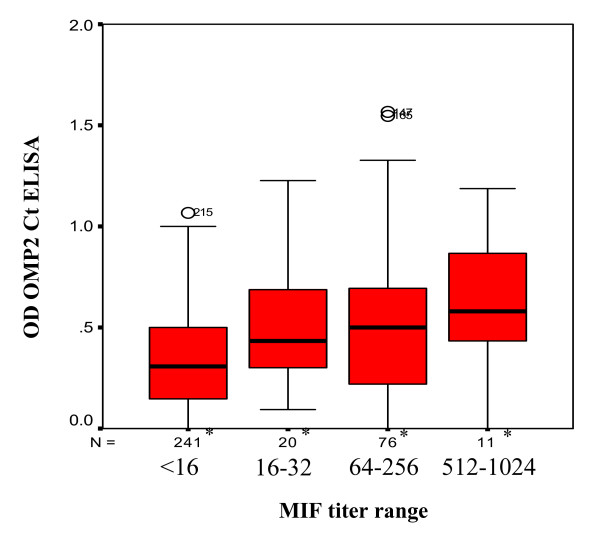
**Correlations between the MIF and OmcB Ct ELISA tests**. *: Number of tested sera at the corresponding MIF titer range. The mean OD values significantly correlated with the MIF antibody titers (p < 0.01).

The distribution of OmcB Ct ELISA indices for all sera, according to the MIF IgG antibody titers, is shown in figure 5. The mean OD values were significantly correlated with the MIF antibody titers (p < 0.01). However, a large distribution of the ELISA indices was found at each MIF titer. Using the optimized cut off value of the ELISA test, the concordance between the MIF and ELISA tests was found to be 73% (259/356). Twenty six sera (7%) were positive by both tests; 233 (66%) negative by both tests; 83 (23%) positive by MIF but not by ELISA and 14 (4%) positive only by ELISA. Using the MIF as the reference method, the OmcB Ct ELISA has a high specificity (94.3%) but a low sensitivity (23.9%) (table 4).

**Table 4:** Performance of the OmcB Ct ELISA relative to the MIF test results.

	**Concordance (%)**	**Sensitivity (%)**	**Specificity (%)**	**PPV^a ^(%)**	**NPV^b ^(%)**
**OmcB Ct ELISA**/**MIF**	72.7 (259/356)	23.9 (26/109)	94.3 (233/247)	65.0	73.7

^a^: PPV, positive predictive value, ^b^: NPV, negative predictive value. For comparison of the OmcB Ct ELISA test relative to the MIF assay, sensitivity, specificity, PPV, and NPV were calculated by using two-by-two tables.

#### Performance of the OmcB Ct ELISA test in selected populations

**Figure 6:**
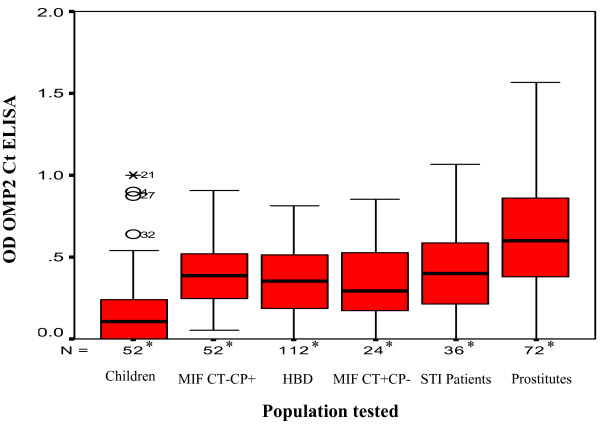
**Performance of the OmcB Ct ELISA in selected populations**. MIF CT-CP+: patients MIF *C. trachomatis *negative *C. pneumoniae *positive, HBD: healthy blood donors, MIF CT+CP-: patients MIF *C. trachomatis *positive *C. pneumoniae *negative, STI patients: patients referred to sexually transmitted infection clinics. *: Number of tested sera according to the population tested. Significant differences were found between the mean OD values of the OmcB Ct ELISA of children's sera as well as prostitutes' sera in comparison with those of the other populations tested (*p *< 0.01). No significant difference was found between the mean OD values of sera from patients MIF *C. trachomatis *positive, patients MIF *C. pneumoniae *positive, patients referred to STI clinics and from healthy blood donors.

The diagnostic value of the OmcB Ct ELISA was also assessed in different populations (table 5). The specificity of the OmcB Ct ELISA test was determined using sera from children, aged from 2 to 8 years, positive sera to *C. pneumoniae *by MIF and sera from patients with negative detection of *C. trachomatis *by PCR and was found to be about 93%. Significant differences were found between the mean OD value of each population and that of children (*p *< 0.01). Furthermore, no significant difference was found between the mean OD values of sera from patients MIF *C. trachomatis *positive, patients MIF *C. pneumoniae *positive, patients referred to STI clinics and from healthy blood donors (fig 6).

**Table 5:** Performance of the OmcB Ct ELISA in selected populations.

**Reference test**	**Population**	**No. of sera**	**ELISA+/ELISA-**	**Specificity (%)**	**Sensitivity (%)**
**MIF**	Children MIF CT-CP-	52	3/49	94.2 (49/52)	NA
	Adulte MIF CT-CP+	52	3/49	94.2 (49/52)	NA
	Patients MIF CT+CP-	24	1/24	NA	4.7 (1/24)

**PCR**	Patients PCR CT-	30	2/28	93.3 (28/30)	NA
	Patients PCR CT+	14	4/14	NA	28.6 (4/14)

NA: not applicable, ELISA positivity: OD ≥ 0.764. Children MIF CT-CP-: children sera MIF *C. trachomatis *negative *C. pneumoniae *negative, patients MIF CT-CP+: patients MIF *C. trachomatis *negative *C. pneumoniae *positive, patients MIF CT+CP-: patients MIF *C. trachomatis *positive *C. pneumoniae *negative, Patients PCR CT-: patients that are *C. trachomatis *PCR negative, Patients PCR CT+: patients that are *C. trachomatis *PCR positive.

The sensitivity of our test was determined using as reference the PCR test in patients *C. trachomatis *PCR positive in whom 4 of the 14 (28.6%) showed antibodies against the OmcB Ct region.

The seroprevalence of *C. trachomatis *antibodies using the OmcB Ct ELISA test in prostitutes and in healthy blood donors was 33% (24/72) and 2% (3/112), respectively. Significant differences were found between the mean OD value of each population and that of prostitutes (*p *< 0.01) (fig 6).

## Discussion

Bioinformatics tools have been reported to be promising in the choice of specific and immunogenic epitopes. In fact, Zhang et al. [41] identified several intertype specific epitopes on human adenovirus hexon using genomic alignment tools, antigenicity and 3-D structure prediction programs. Most of the predicted epitopes were found in the prepared synthetic peptides and recombinant proteins. In another study, Xu *et al*. [42] obtained a high titer and a good specificity of the anti-mouse AdipoR-1 polyclonal antibodies, following immunization using the selected polypeptide by bioinformatics analysis through the Antigenic, the AntigenProfiler and the ClustalW programs. In the same context, we evaluated the potential utility of the *in silico *methods for the prediction of specific and immunogenic regions in the OmcB protein of *C. trachomatis*. We used the ClustalW computer program, which generates multiple sequence alignment, in order to diminish the extent of cross reactions frequently seen with antibodies raised against the OmcB protein of the chlamydial species. This program allowed us to detect two regions in the OmcB protein, localized in the Nt and Ct regions, with high homology within *C. trachomatis *serovars and low homology with those of the other chlamydial species infecting humans. Mygind *et al*. [33] reported that in contrast to the Nt region of the OmcB protein covering approximately 70 amino acids which is highly variable among chlamydial species [31] showing homology values ranging between 19 and 28%, the Ct region spanning amino acids 87 to 547 is highly conserved.

Synthetic peptides mimicking epitopes, as well as antipeptide antibodies, have many applications in the diagnosis of various human diseases [43-45]. During the last 25 years, immunoinformatic software and databases for B cell epitope prediction have been developed to facilitate epitope design that could be synthesized and used instead of the antigen. These programs focused essentially on linear epitope prediction and were based upon various amino acid properties including beta turn prediction [46], surface accessibility [47], flexibility [48] and hydrophilicity [49]. In the current study, we have used the Antigenic program of the Pasteur institute which is based on amino acid frequencies in antigenic domains and chemistry, to predict potentially immunogenic regions in a protein [36]. The application of this method to a large number of proteins allowed an accuracy of 75%. The immunogenicity prediction according to the full length OmcB protein indicates that the epitopes are distributed throughout the protein and that the OmcB Ct region appears more immunogenic than the Nt region. The latter was previously found to be of limited immunogenicity in experimentally immunized rabbits [31] as well as in humans, and thus of little serodiagnostic use [33]. Whilst Mygind *et al*. [33] failed to identify a species specific B cell epitope in the OmcB protein of *C. trachomatis*, Goodall *et al*. [50] described a species specific *C. trachomatis *T cell epitope spanning amino acids 400 to 413 overlapping in 10 of its 14 amino acids with the Ct region identified in our study.

The Nt and Ct regions selected in this study were cloned into two expression vectors in order to test their ability to better express these regions. We also used different tags to identify which of them generates less reactivity with human sera. The expression level of the Nt region using the two vectors was too low and the presence of the Nt recombinant proteins was not reproducible. Recently, an important decrease in the expression levels of the GST and GST-fused proteins in both native and recombinant pGEX vectors has been explained by the occurrence of a spontaneous deletion of 701 bp leading to the loss of the tac promoter directing transcription [51]. In our study, a strong degradation was noticed in the proteins expressed using the PinPoint Xa-1 vector despite the presence of protease inhibitors. The production of untagged biotinylated proteins was also reported even after careful optimization of the culture conditions [52,53]. In contrast to the Nt region, the Ct region was well expressed using the two vectors. Differential expression was noticed only with the BPT-Ct as JM109 *E. coli *cells exhibit a tenth less expressed protein than *E. coli *BL21 (DE3) (data not shown). As opposed to the GST-Ct and to the GST, we could not purify the BPT-Ct and the BPT by affinity chromatography using different protocols [52,54]. Surprisingly, despite the instability of the biotinylated fusion proteins, they have been found to be heat treatment resistant at 70°C for 5 minutes. Increasing temperature or incubation time gives minimal yield of these two proteins without eliminating thermo-resistant contaminants (data not shown). This property is buffer dependent and is probably related to the amino acids content of these proteins. Immunogenicity of the Ct region was first investigated by western blot analysis that revealed discrepancies between the BPT-Ct and GST-Ct results. These discrepancies were related to the tag reactivity that was greater using the PinPoint Xa-1 vector than using the pGEX-6P-1 vector. Only a few reports described the reactivity of biotinylated proteins with human sera in which the biotinylated tag interfere with the developed ELISA assays [53,55]. Different authors did not notice any reactivity of the GST with human sera [29,56,57]. Moreover, although GST derived from *Schistosoma japonicum*, the use of recombinant GST tagged protein detected only one of 88 sera from patients with schistosomiasis [58]. In our study, a strong reactivity of the GST was seen especially by ELISA in line with previous reports [59,60]. Thus, we used the GST-Ct as antigen and the GST as control in the ELISA as they were purified to homogeneity and as the GST exhibits less reactivity with human sera.

Several authors studied the humoral immune response to the OmcB protein of the chlamydial species. Klein *et al*. [29] performed a comparative serological analysis of several surface antigens of *C. trachomatis *and *C. pneumoniae *in immunoblot assays and noticed that the strongest and most frequent reactions were observed towards the recombinant OmcB proteins of both species. All MIF *C. pneumoniae *positive sera reacted with the OmcB protein from both species, whereas, MIF *C. trachomatis *positive sera displayed OmcB reactivity at considerably less frequency but with a significant degree of cross reaction with antibodies to the OmcB of *C. pneumoniae*. In another study, Mygind *et al*. [33] found that 95% of MIF *C. trachomatis *positive sera and 80% of MIF *C. pneumoniae *positive sera showed significant antibody titers against *C. trachomatis *OmcB protein. Bas *et al*. [32] evaluated whether new ELISA assays could improve the serological diagnosis of *C. trachomatis *reactive arthritis and reported that the highest sensitivity and the lowest specificity were obtained with the OmcB protein that were 100% and 45%, respectively. This low specificity was related to the antibodies' cross reactivity as 42% of the patients had IgG antibodies' to *C. pneumoniae*. Portig *et al*. [61] developed ELISA tests by using recombinant OmcB proteins from both species and reported that 75% of patients with urethritis and those from a Gambian endemic region and 100% of patients tested with active trachoma and patients with sexually acquired arthritis were OmcB *C. trachomatis *positive. The specificity of the OmcB protein was reported to be low as the antibody titers were higher on the homologous protein in 76% of cases.

Using our developed ELISA test, a sensitivity of only 23.9% and a specificity of 94.3% were found relative to the MIF test indicating that the use of the sequence alignment tool might be a useful method for identifying species specific regions in an immunodominant antigen. However, the two epitopes, located in the Ct region, of the 24 predicted in the full length OmcB protein account for approximately 25% of the serological response detected by MIF limiting the use of the developed ELISA test when screening *C. trachomatis *infections.

When using selected populations, the same specificity was found in children and in patients *C. pneumoniae *MIF positive. However, the mean OD value found in MIF *C. pneumoniae *positive sera was in the same range as those from patients referred to STI clinics, from patients MIF *C. trachomatis *positive and from healthy blood donors. The same results were obtained by Mygind *et al*. [33] who reported that 80% of *C. pneumoniae *MIF positive sera showed antibodies to *C. trachomatis *but did not show lower amount of antibodies to the OmcB protein.

Only 2 of the 30 (7%) patients with *C. trachomatis *PCR negative results were OmcB Ct ELISA positive. One of them was found *C. trachomatis *PCR positive in the latest sixth months indicating previous infection with the pathogen leading to persistent antibodies. Indeed, IgG antibodies to *C. trachomatis *have been described to persist over many years although titers decline in some situations [62,63]. Furthermore, only 4 of the 14 (28%) patients *C. trachomatis *PCR positive were OmcB Ct ELISA positive. This low sensitivity detected in PCR positive patients is probably due to the delayed seroconversion since the production of antibodies occurs almost 9 to 32 days following initiation of symptoms [64,65]. Portig *et al*. [61] compared sera from patients with suspected *C. trachomatis *infections whose vaginal or urethral swabs tests were negative or positive and found that positivity to the *C. trachomatis *OmcB antibodies was 15% and 75%, respectively. In another study, Bas *et al*. [32] compared the performance of the OmcB protein to other chlamydial proteins in patients with acute *C. trachomatis *infection and reported that this protein showed the highest sensitivity (89%) but the lowest specificity (57%).

## Conclusion

The results of our study demonstrated that recombinant proteins should be used with caution since the vector tags reactivity with human sera is frequently seen. Furthermore, the *in silico *prediction approach led to a high specificity by means of the developed OmcB Ct ELISA test, however; its low sensitivity limit the use of the developed ELISA when screening *C. trachomatis *infections.

## Authors' contributions

Author OFG performed bioinformatics research, carried out laboratory experiments, the evaluation of the ELISA, analyses of data, and drafted the manuscript. Author RG participated in the bioinformatics research, analysis of data and coordination of the study. Author AZ participated in the analysis of data and coordination of the study. Author BG participated in the bioinformatics research and cloning. Author JG provided the healthy blood sera and coordinated the study. Author AR provided bioinformatics help and coordinated the study. Author AH participated in design, data analyses, and coordination of the manuscript study. All the authors read and approved the final manuscript

## Supplementary Material

Additional file 1 **Antigenicity prediction of the OmcB protein of *****C. trachomatis***. The data provided represent the epitope location in the Nt or the Ct region in the OmcB protein, the epitope classification and position according to the full length OmcB protein as well as the epitope sequence and length. The residue in bold is the amino acid with the highest score in the epitope. The antigenicity prediction was performed using the Antigenic program according to Genbank accession number [EMBL:CAA39396]. Click here for file
