# Supplementary material for: Evaluation of an in silico predicted specific and immunogenic antigen from the OmcB protein for the serodiagnosis of Chlamydia trachomatis infections
Source: BMC Microbiol. 2008 Dec 10;8:217. doi: 10.1186/1471-2180-8-217 (PMC2615015; doi:10.1186/1471-2180-8-217)
Supplement: Additional file 1 — Antigenicity prediction of the OmcB protein of C. trachomatis. The data provided represent the epitope location in the Nt or the Ct region in the OmcB protein, the epitope classification and position according to the full length OmcB protein as well as the epitope sequence and length. The residue in bold is the amino acid with the highest score in the epitope. The antigenicity prediction was performed using the Antigenic program according to Genbank accession number [EMBL:CAA39396]. [file 1471-2180-8-217-S1.doc]

| **Epitope location ^a^** | **Epitope classification ^b^** | **Epitope sequence ^c^** | **Epitope position ^d^** | **Epitope Lengh** | **Epitope score** |
| --- | --- | --- | --- | --- | --- |
|  | 1 | NACLR**C**PVVYKINVVNQG | 220-237 | 18 | 1.254 |
|  | 2 | PGDLVLR**D**VVVEDT | 342-355 | 14 | 1.217 |
| **Ct** | 3 | NNVVV**K**SCSDCGTCTSCAEA | 404-423 | 20 | 1.216 |
|  | 4 | WKGVAATHMCVVDTCD**P**VCVGEN | 427-449 | 23 | 1.204 |
|  | 5 | VTTVINEPCV**Q**VSIA | 308-322 | 15 | 1.203 |
| **Ct** | 6 | SLQY**K**VLVRAQ | 387-397 | 11 | 1.196 |
|  | 7 | WSYV**C**KPVEYVISVS | 326-340 | 15 | 1.192 |
|  | 8 | KITVWVKPLKEGCCFTAATVCA**C**PEIRSVTKCGQPAICVKQ | 175-215 | 41 | 1.191 |
|  | 9 | VY**R**ICVTSR | 451-459 | 9 | 1.182 |
|  | 10 | ARNVVV**E**NP | 241-249 | 9 | 1.175 |
|  | 11 | IAT**V**SYCGG | 293-301 | 9 | 1.156 |
|  | 12 | NTVVF**D**SLPRL | 493-503 | 11 | 1.150 |
|  | 13 | SPGVTVLEAAGAQISC**N**KVVWTV | 357-379 | 23 | 1.150 |
| **Nt** | 14 | RRAVT**I**FAVTSVASLFASGVLET | 6-28 | 23 | 1.149 |
|  | 15 | TITVEF**C**PLK | 277-286 | 10 | 1.140 |
|  | 16 | CAD**V**IITQQLPCEAEFVRSD | 131-150 | 20 | 1.134 |
|  | 17 | TVEFSVTL**K**AVSAG | 508-521 | 14 | 1.133 |
|  | 18 | EAILSSDTLT**V**PVS | 526-539 | 14 | 1.130 |
| **Nt** | 19 | ETLVDRKEVAP**V**HES | 64-78 | 15 | 1.123 |
|  | 20 | SKELQP**V**SFS | 474-483 | 10 | 1.118 |
|  | 21 | ITQAVPE**Y**ATVGSPYPLEIT | 105-124 | 20 | 1.116 |
|  | 22 | NVSL**M**LK | 466-472 | 7 | 1.106 |
|  | 23 | GQRVLT**F**T | 259-266 | 8 | 1.095 |
| **Nt** | 24 | TNVIS**L**ADT | 36-44 | 9 | 1.091 |
|  | 25 | PD**G**YAHS | 251-257 | 7 | 1.074 |
|  | 26 | GKL**V**WKI | 159-165 | 7 | 1.059 |

The antigenicity prediction was performed using the Antigenic program. ^a^: Epitope location in the N-terminal (Nt) or the C-terminal (Ct) region in the OmcB protein, ^b^: Epitope classification according to the antigenicity prediction of the OmcB protein, ^c^: Sequence of the epitope, the residue in bold is the amino acid with the highest score in the antigenicity prediction, ^d^: Epitope position in the full lengh OmcB protein.
